# Community Mobilisation and Empowerment Interventions as Part of HIV Prevention for Female Sex Workers in Southern India: A Cost-Effectiveness Analysis

**DOI:** 10.1371/journal.pone.0110562

**Published:** 2014-10-21

**Authors:** Anna Vassall, Sudhashree Chandrashekar, Michael Pickles, Tara S. Beattie, Govindraj Shetty, Parinita Bhattacharjee, Marie-Claude Boily, Peter Vickerman, Janet Bradley, Michel Alary, Stephen Moses, CHARME India Group, Charlotte Watts

**Affiliations:** 1 London School of Hygiene and Tropical Medicine, London, United Kingdom; 2 St John's Research Institute, Bangalore, India; 3 Imperial College, London, United Kingdom; 4 Karnataka Health Promotion Trust, Bangalore, India; 5 URESP, Centre de recherche du CHU de Québec, Québec, Canada; 6 Département de médecine sociale et préventive, Université Laval, Québec, Canada; UNAIDS, Trinidad And Tobago

## Abstract

**Background:**

Most HIV prevention for female sex workers (FSWs) focuses on individual behaviour change involving peer educators, condom promotion and the provision of sexual health services. However, there is a growing recognition of the need to address broader societal, contextual and structural factors contributing to FSW risk behaviour. We assess the cost-effectiveness of adding community mobilisation (CM) and empowerment interventions (eg. community mobilisation, community involvement in programme management and services, violence reduction, and addressing legal policies and police practices), to core HIV prevention services delivered as part of *Avahan* in two districts (Bellary and Belgaum) of Karnataka state, Southern India.

**Methods:**

An ingredients approach was used to estimate economic costs in US$ 2011 from an HIV programme perspective of CM and empowerment interventions over a seven year period (2004–2011). Incremental impact, in terms of HIV infections averted, was estimated using a two-stage process. An ‘exposure analysis’ explored whether exposure to CM was associated with FSW’s empowerment, risk behaviours and HIV/STI prevalence. Pathway analyses were then used to estimate the extent to which behaviour change may be attributable to CM and to inform a dynamic HIV transmission model.

**Findings:**

The incremental costs of CM and empowerment were US$ 307,711 in Belgaum and US$ 592,903 in Bellary over seven years (2004–2011). Over a 7-year period (2004–2011) the mean (standard deviation, sd.) number of HIV infections averted through CM and empowerment is estimated to be 1257 (308) in Belgaum and 2775 (1260) in Bellary. This translates in a mean (sd.) incremental cost per disability adjusted life year (DALY) averted of US$ 14.12 (3.68) in Belgaum and US$ 13.48 (6.80) for Bellary - well below the World Health Organisation recommended willingness to pay threshold for India. When savings from ART are taken into account, investments in CM and empowerment are cost saving.

**Conclusions:**

Our findings suggest that CM and empowerment is, at worst, highly cost-effective and, at best, a cost-saving investment from an HIV programme perspective. CM and empowerment interventions should therefore be considered as core components of HIV prevention programmes for FSWs.

## Introduction

The UNAIDS strategic investment approach for an effective response to HIV/AIDS proposes a package of basic programme interventions, including HIV prevention for key populations [1], [2]. Traditionally, core HIV prevention interventions for female sex workers (FSWs) are individual behaviour change involving peer educators, condom promotion and the provision of sexual health services [3], [4]. However, there is a growing recognition that wider social and community level factors influence an individual’s ability to adopt safer sexual behaviours [5]. As a result, many actors working in HIV prevention argue it is necessary to simultaneously address the broader societal and contextual factors that may contribute to HIV-related risk behaviour [6]–[12] as an essential element in achieving and sustaining the impact and efficiency of HIV prevention services targeted at FSWs [2].

Within the UNAIDS investment framework, ‘critical enablers’ are defined as activities that are necessary to support the effectiveness and efficiency of basic programme activities [13]. For FSW-targeted HIV prevention these community mobilisation (CM) and empowerment interventions may include: community mobilisation, community involvement in programme management and services, violence reduction, and addressing legal policies and police practices. These interventions are argued to improve programme effectiveness through the ‘empowerment’ of FSWs, with empowerment defined as ‘the *process* by which those who have been denied the ability to makes choices (the disempowered) acquire such an ability’[14] - in this case the aim being to empower individual FSWs to negotiate safer sex and access services, as well as address other issues affecting their lives (such as violence or poverty), supported by the social solidarity of the broader community of FSWs [15].

There are a number of studies on the cost-effectiveness of HIV prevention targeted at FSWs [16]–[18]. For example, a recent study, modelling the potential cost-effectiveness of FSW-targeted HIV prevention in India (including impact on the general population), found an incremental cost per disability adjusted life year (DALY) averted of US$ 10 [19] for a basic package of services. Several further studies also suggest that cost-effectiveness can be achieved in practice and at a scale [20]–[22]. However, despite this clear evidence that investment in HIV prevention for FSWs is highly cost-effective in concentrated epidemic settings, the ‘added value’ of investing in the mobilisation and empowerment of FSWs as part of the HIV prevention package for key populations remains unclear. This lack of evidence can provide a substantial constraint to those seeking funding to implement comprehensive approaches to supporting FSWs, when funders are seeking ‘value for money’ and are scrutinising any cost that may be seen as ‘non-essential’. Given the growing evidence that CM and empowerment improve programme impact [23]–[27], without sound evidence of cost-effectiveness there is a risk that the gains that have been made to date by more comprehensive HIV prevention programmes for FSWs [28], [29] will be jeopardised.

We therefore present here a cost-effectiveness analysis of CM and empowerment interventions, when added to the core package of HIV prevention for FSWs, as part of the *Avahan* Programme in two districts of Karnataka state, South India, in order to inform funders, policy makers and programme managers designing HIV prevention for FSWs globally.

## Methods

### Study setting and population studied

It is estimated that 2.27 million people are living with HIV in India [30]. The epidemic is concentrated, and predominately driven by marginalised groups, particularly FSWs, men who have sex with men (MSM), and in some contexts injecting drug users [31], [32]. The *Avahan* programme, the India AIDS Initiative of the Bill & Melinda Gates Foundation (BMGF), is one of the largest HIV prevention programmes targeted at high risk groups in the world. The programme operates across six Indian states and had a funding commitment of US$ 258 million between 2004 and 2009 [33], and thereafter was transitioned to national ownership. *Avahan* is implemented through contracted state lead partners (SLPs), who in turn contract a plethora of local non-governmental organisations (NGOs) at the district level.

The state of Karnataka is one of the four southern states in India supported by *Avahan*, with an estimated HIV adult prevalence of 0.9% (2008), and it contributes 11% of the HIV burden in the country [30]. Unprotected heterosexual sex is a key factor in the spread of the epidemic, with overall prevalence among FSWs in the state being 16.4% [32]. The *Avahan* SLP in HIV prevention efforts is the Karnataka Health Promotion Trust (KHPT), supported by the University of Manitoba - and has worked with 17 NGOs across 19 districts since 2004. Our cost-effectiveness analysis focuses on two districts in Karnataka, Belgaum and Bellary. These two districts were chosen purposively, based on Karnataka’s socio-cultural regions, the size of the high-risk population and the availability of detailed behavioural, biological and costing data. Table 1 shows the number of FSWs reached at least once a year. For Belgaum this ranges from 806 in 2004 to 3072 in 2011; and in Bellary from 2741 in 2004 to 5743 in 2011.

**Table 1:** HIV prevention core and community mobilisation and empowerment services provided by year (US$ 2011).

	2004/5	2005/6	2006/7	2007/8	2008/9	2009/10	2010/11
***Belgaum***							
FSW population contacted at least once a year	806	2,776	3,143	4,237	2,294	2,679	3,072
Drop-in Centres	1	8	15	17	17	16	16
FSW peer groups			18	186	233	163	117
FSW members of peer groups			250	2,328	2,609	2,155	1,607
FSWs attendances to committee meetings			582	4,597	19,481	20,712	13,697
***Bellary***							
FSW population contacted at least once a year	2,741	3,066	4,464	4,157	4,341	5,055	5,743
Drop-in Centres	0	7	14	14	13	14	14
FSW peer groups				158	168	168	168
FSW members of peer groups				2,364	2,568	2,568	2,568
FSWs attendances to committee meetings				25,529	25,264	34,541	38,450

Data from routine management data sources (Avahan Comprehensive Management Information System).

### Intervention description

The package of core HIV prevention services provided by the Avahan NGOs in both districts includes outreach through peers, behaviour change communication, condom distribution, and clinical services for sexually transmitted infections (STIs). Peer educators provided services to about 25–50 people each, sharing prevention information, distributing supplies (condoms and lubricants) and providing referral for STI management. Referral clinics followed standard protocols for STI management. There was active referral of individuals for HIV testing and positive key populations were referred to government anti-retroviral treatment (ART) centres for care and support. This core package has been demonstrated as cost-effective [34].

CM services and actions to create an ‘enabling environment’ were added to these core services in order to empower FSWs. Full details of the intervention are also publically available [35], [36]. In the initial stages, activities concentrated on drop-in centres (DICs) as a focal point for community mobilization (2004–6). DICs provided a platform to motivate FSWs to use HIV prevention, but also drew in FSWs by holding special events. In rural areas, at the village level, FSW peer groups were established as a platform for FSWs to share and discuss issues and develop income generation activities (2006–07). FSW peer groups aimed to create awareness on HIV/AIDS, but also address non-health needs such as helping to access government welfare schemes including ration cards, education for the children of FSWs, etc. From 2008 onwards, FSW peer groups were strengthened through the formation of district-level collectives; and over time these provided the basis to form community-based organizations that could take on an active role in programme implementation and management. Similarly, building on the connections made in the DICs, FSWs were further encouraged to be involved in the planning of HIV prevention services through the formation of various programme committees.

In addition to the formation of communal structures, broader efforts to create an ‘enabling environment’ were taken to improve the FSWs’ interaction with the wider community, further contributing towards their empowerment. This included conventions to encourage community members to interact with other FSWs in different villages and the provision of legal empowerment sessions [12]. There were also sensitization sessions for local leaders, youth organizations and secondary stakeholders like pimps, brothel owners, police and others – particularly focused on violence reduction [11]. All the activities were supported through capacity building from the SLP and its supporting partners. Table 1 shows the numbers of DICs, FSW peer groups and FSW membership and attendances to committee meetings in each district.

### Intervention effectiveness

We have recently conducted a study [37], [38] examining the impact of CM on FSW empowerment, risk behaviours and HIV/STI prevalence in four districts in Karnataka. This study used data from serial cross-sectional bio-behavioural surveys, termed integrated behavioural and biological assessments (IBBAs), that were carried out amongst FSWs and clients in districts in Karnataka state in 2008 (3 years after programme initiation) and in 2011 (further details below). Detailed results of this exposure analysis are presented elsewhere [37], [38], and a summary of the methods and results used can be found in Appendix S1 (Tables S1 and S2).

We used a conceptual hierarchical framework of protective factors for HIV and STI infection to explore the possible pathways through which CM may impact key HIV related outcomes, over and above peer education activities alone [37]. By 2011, for all measures of power, HIV/STI service uptake and condom use, adjusting for CM, caused the adjusted odds ratio (AOR) (for peer education and these HIV related outcome variables) to shift substantially closer to 1.0, suggesting CM was having a key mediating effect on the pathway between peer education and these HIV related outcomes (Table S2 in Appendix S1). When peer education was significantly associated with an outcome variable, we then calculated the proportion difference in the adjusted ORs between peer education and the HIV related outcome variable, and the adjusted ORs with the adjustment for CM (OR adjusted for confounders – OR adjusted for confounders and CM)/OR adjusted for confounders) (last column of tables S1–S2). In particular, and for the purposes of this study, the AOR for peer education and reported condom use at last sex with occasional clients changed from 10.96 (5.00, 24.01) (p<0.001), to 6.78 (1.96, 23.44) (p = 0.003), when we included CM in the model, representing a proportional reduction in the AOR of 38.1% in 2010/11. Similar results were also seen in baseline (Table S1 in Appendix S1), where the proportional reduction in the AOR was found to be 46.2%.

### Framework for cost-effectiveness analysis

Drawing upon these findings, we estimate the incremental (additional) costs and impact of CM and empowerment activities described above compared to a base case of providing just the core HIV prevention activities for FSWs from an HIV programme [39] (provider) perspective for a seven year period (2004–2011). We estimate two measures of incremental cost-effectiveness, incremental cost per HIV infection averted (within the seven year period), and incremental cost per DALY averted, (DALYs averted from HIV infections averted within the seven year period over the potential lifetime of FSWs). The latter is compared to a World Health Organisation (WHO) defined willingness to pay (WTP) threshold of Gross National Income (GNI) per capita (US$ 1410) for India in 2011 [40], [41] to assess cost-effectiveness. A discount rate of 3% is used in our primary estimates for both costs and DALYS.

#### Incremental cost estimation

An ingredients approach was used to estimate incremental economic costs prospectively from the perspective of the HIV programme. The UNAIDS ‘Costing Guidelines for HIV Prevention Strategies’ were taken as the basis for the costing methodology [42]. Costs were collected from every organizational level that was involved in the provision of services: NGO, SLP, the BMGF office in Delhi (including pan-*Avahan* capacity building costs).

Cost data were obtained from routine financial and management reporting, as well as from staff records and interviews with staff. Details of donated goods and services were collected from each district. The economic costs of donated items were valued at market prices. Capital costs were annualised using a standard discount rate of 3% [43], [44]. Start-up and training costs were annualised over the first five years. The start-up period was defined as district-level inception until the start of service delivery to the target population.

Personnel costs covered the salaries and expenses of all staff, including peer educators, volunteers and shared resource personnel. Peer educator time was valued at the honorarium paid, except when not paid. In the latter case, and for other volunteers, time costs were valued using self-reported average earnings or, if unemployed, the payment made to peers in interventions undertaken by the National Aids Control Organisation (NACO), Government of India. Condom costs were estimated using the lowest-priced market alternative.

All costs that were not spent on a specific use were allocated to different programme activities on the basis of time sheets (personnel-related costs) or interviews with staff (use of capital items), supported by programme activity reports. BMGF and pan-*Avahan* capacity building costs were allocated to SLPs according to the size of the grant during the year of analysis. SLP costs that could not be allocated for a specific use were broadly allocated according to either interviews with staff, on an equal basis for costs associated with programme management, or using estimated population for activities directly related to service provision.

Incremental costs for CM and empowerment activities were calculated for the numbers of FSWs reached per year. The number of people contacted at least once in a year reflects programme reach and was measured using programme management information system (MIS) data. All data were entered into a specifically designed MS Excel workbook and converted to US$ 2011 prices using the GDP deflator index [45] and the mid-year exchange rate for 2011 [43].

#### Incremental impact estimation

Incremental impact, in terms of HIV infections averted, was estimated using a two-stage process drawing on the exposure analysis. The exposure analysis, described above was used to estimate the incremental changes in HIV-related sexual risk behaviour related to exposure to the CM and empowerment intervention in Bellary and Belgaum. In the second stage, the results of this exposure analysis were then inputted into a dynamic transmission model that estimated the number of HIV infections averted from the incremental changes in HIV-related sexual risk behaviour relative to a simulated control scenario where no CM and empowerment activities were present.

### Survey data used

As part of the evaluation of *Avahan,* IBBAs surveys were carried out amongst FSWs and clients in districts in Karnataka state. Programmes were initiated between April 2004 and July 2005, with the IBBA surveys in FSWs conducted in 2005/6, 2008 and 2011. Client IBBA surveys were carried out in October 2007 in Belgaum and Bellary. Sample size calculations and the sampling methodology have been described in detail previously [46], with approximately 400 respondents randomly sampled at each round in most districts, using a probability-based sampling method. Appropriate weights were used to account for the differential non-response rates and differential probabilities of selection across districts, as well as the differential recruitment of FSW by typology within districts.

In addition to questions on sexual behaviour and risk factors for HIV, blood samples were taken to test for HIV, HSV-2 and syphilis [47]. IBBA survey data were subsequently used in the exposure analysis and to calibrate the dynamic HIV transmission model.

### Exposure analysis

Following discussions with project staff, we used the following data from the IBBAs as proxy measures to define exposure to CM activities: “high” if a woman reported being a member of a FSW peer group or FSW collective; “medium” if she had ever attended a drop-in center or a nongovernmental organization (NGO) meeting; and “low” if she had not done any of the preceding. Measures of collective power (power with) were explored using the following questions: “I feel a strong sense of unity with FSWs I do not know”; “In the last 1 year, have you negotiated with or stood up against a powerbroker to help a fellow FSW?”; and “In the last 6 months, have you attended any public events, such as a rally or a gathering of sex workers, where you could be identified as a sex worker?”. A measure of individual power (power to) was developed by looking at whether respondents had answered “no” to “In the past 1 month, was there a time when you wanted to use a condom during sexual intercourse but did not use it?” or “In the past 1 month, was there a time when you wanted to use a condom during sexual intercourse with a client but did not use it?”

For the exposure analysis, we used the results from this analysis [37] derived from data from the 2005/6 and 2011 IBBA surveys for FSWs pooled across four districts (Bangalore and Shimoga in addition to Belgaum and Bellary) and applied them to Belgaum and Bellary. This was justified because the implementation of CM and empowerment was standardized across *Avahan* districts, and the district-pooled analysis was necessary to have sufficient unexposed individuals to CM and empowerment activities (incremental to the broad HIV programme). The effect size (proportional AOR) derived from the exposure analysis was assumed to vary over time, starting at the 2005/6 value (46.2%) and then increasing linearly over time to the value at 2011 (38.1%), after which it was taken to be constant. This effect size was then incorporated in the dynamic HIV transmission model described below.

It should be noted that the examination of effect did include controls for socio-demographic variables. District, duration in sex work, sex work typology (place where solicit clients or place where have sex with clients), relationship status (marital status or regular partner), and socio-economic status (literacy or additional income to sex work) were added a priori. The adjusted Wald test was used to test for effect modification for the following socio-demographic variables: age, duration in sex work, place where solicit clients, place where have sex with clients, charge per sex act, and regular partner.

### Impact model

A purpose-built dynamic compartmental model of HIV/STI transmission was then used to calculate HIV infections averted in FSWs and their clients, as well as their partners in the general population, over the first seven years of the *Avahan* programme [48]. Further details of the model are publically available [29], [49]. Within the model, the basic package of services is assumed to have two effects: increasing FSW condom use with clients, and enhancing STI treatment in clinics. Other intervention components such as structural interventions, and CM and empowerment, are considered to act indirectly on HIV transmission through increasing condom use. The effect estimates from the causal pathway model for condom use at last sex with occasional clients (see Tables S1 and S2 in Appendix S1) were used to parameterise the fraction of increases in FSW condom use with clients associated with the CM and empowerment component of the intervention, and thus explore the impact of CM and empowerment activities on HIV infections averted using the simulated matched control groups described below. Unlike most previous exercises in modelling impact for cost-effectiveness analyses at scale in India [19], [21], [22], the model used is dynamic (incorporating indirect population-level effects not captured in cohort models) and is employed within a Bayesian framework [1], [29] to allow a better quantification of uncertainty.

Each district was parameterized separately, using behavioural parameters derived from the relevant district FSW and client IBBA surveys. For each parameter a range was defined using the 95% confidence interval of the survey data. Ranges for biological parameters such as per-act HIV transmission probabilities were obtained from extensive reviews of published literature, and the same parameter ranges were used for both districts (see Table S3 in Appendix S1 which summarises the key model parameter ranges and references). A full list of parameters, along with a detailed description of the model equations and structure, is also publically available [29], [49]. All parameter ranges were sampled extensively through Latin hypercube sampling (LHS) to generate combinations of parameters for each district. Enough combinations of parameters were generated through LHS to fully account for parametric uncertainty in each district, with one million and 100,000 parameter sets sampled for Belgaum and Bellary districts respectively.

In a previous evaluation of the overall HIV impact of *Avahan* [29], [49] the *intervention* scenario in which condom use increased as given by trends in IBBA survey data was compared against a control scenario describing how condom use might have changed without the intervention (i.e. a first matched simulated control group). For the intervention scenario, condom use over time in each district was estimated using a historical cohort method [50]. Condom use increased at a slower rate before *Avahan*, and then increased sequentially up to the level of each FSW IBBA survey, remaining constant after the last round. The model was then run under the *intervention* scenario with each parameter set as an input to obtain “fits” to the confidence intervals of the first round of local HIV/STI prevalence derived from IBBA survey data for FSWs and clients, as well as the FSW HIV prevalence ratio between Round 1 and subsequent rounds.

In the *control* scenario, condom use was assumed to have increased during *Avahan* at the pre-intervention rate, while STI treatment was assumed to continue at pre-intervention levels. The difference between the *intervention* scenario and evaluation *control* scenario thus gives the increase in condom use due to *Avahan* over time. This incremental effect is used to generate our primary estimates for the impact and cost-effectiveness analysis of the entire *Avahan* package of services.

The effect size of CM and empowerment on FSW condom use, which was taken to be the same in both districts, was used in a similar way to define a second simulated control group. This second simulated control group, with no exposure to CM and empowerment activities, was created by assuming that a fraction of the total increase in condom use due to *Avahan*, as defined above, was due to CM and empowerment activities, as estimated through the exposure analysis. Removing this fraction created a *no-CM and empowerment (no-CME) control* group scenario. The multiple parameter combinations which were found to be “fits” to survey prevalence data under the intervention scenario were re-run for the *no-CME control scenario* to obtain the number of new HIV infections without the CM and empowerment component of the intervention. By comparison with the number of HIV infections occurring in each of the corresponding original “fits” from the intervention scenario, estimates for the incremental infections averted over 7 years by CM and empowerment were obtained, with the multiple “fits” producing an uncertainty range. DALYs averted were estimated from incremental infections averted generated by the impact model, using standard formulae and disability weights [41], a 3% discount rate and no age weighting. Table S4 also includes the parameters used to estimate DALYS.

### Sensitivity Analyses

We conducted a probabilistic sensitivity analysis randomly sampling cost parameters with 5,000 iterations to estimate mean incremental costs per DALY averted. The parameters sampled at this stage were annual NGO, SLP and BMGF costs, age at HIV infection (based on IBBA data), and DALY weights (see Table S4 in Appendix S1 for details). In addition, the impact model “fits” were also sampled uniformly at the same time, corresponding to different impacts of CM and empowerment.

We also conducted a one-way analysis to examine how our results may change if antiretroviral therapy (ART) was available. We did not include prevention effects from ART, as ART was not provided in a widespread manner during the time of intervention, but rather explored the likely impact in terms of the future cost savings associated with reductions in the need for ART from infections averted. This was done using the dynamic HIV transmission model to predict the number of individuals who would have started ART by 2011 assuming future coverage rates of 21–40% of people meeting the eligibility criterion CD4<350 cells/mm^3^. For the sensitivity analysis, coverage of ART, the incremental increase in life expectancy from ART, and the annual unit cost of ART were all sampled as well. We also conducted a further sensitivity analysis using discount rates of 0%, 5% and examined the probability that any incremental cost-effectiveness ratios were below the WTP threshold using an acceptability curve, which shows the probability of the incremental cost effectiveness being below the WTP taking into account the parameter uncertainty.

### Ethical approval

The estimates of the effect of CM and empowerment come from the secondary analysis of IBBA data in Karnataka. Participation in the IBBAs was by witnessed, verbal, informed consent. Only women aged 18 years or more were recruited in the study. The surveys were conducted anonymously, with no names or personal identifiers recorded. A detailed and standardized consent process was implemented for each respondent, and consent was obtained separately for the interview and for giving biological samples. Participants’ consent was provided in writing, either by themselves, or for illiterate participants, by an independent witness who confirmed that verbal consent was provided. Statutory approval for the conduct of the IBBAs and their protocols was obtained from the Government of India’s Health Ministry Screening Committee (HMSC). The study was approved by the Institutional Ethical Review Board of St. John’s Medical College, Bangalore, India, and the Health Research Ethics Board of the University of Manitoba, Winnipeg, Canada.

## Results

### Costs

Community mobilisation and empowerment activities cost between US$ 31,690–US$ 64,304 in Belgaum and US$ 53,349–US$ 135,572 in Bellary per year (Table 2). Total costs rise initially then fall after the first four to five years of the intervention, broadly in line with the pattern of total HIV prevention costs. Overall HIV prevention costs increased until 2007/8 as the programme reached out to more people (also see Table 3). In 2008 the programme began to transition to national ownership and fell back dramatically. Total CM and empowerment costs increased from the first to second year, due to the increased ability of NGOs to expand CM and empowerment once the rapport with the community was built. In the later years, post 2008, as with overall prevention costs, CM and empowerment costs start to decrease due to preparation for transition in funding to local state HIV programmes. Reductions in costs may also reflect improvements in efficiency with programme maturity and scale [51].

**Table 2:** Incremental community mobilisation (CM) and empowerment costs by organisational level and year (US$ 2011) and CM and empowerment as a percentage of total HIV prevention costs.

	2004/5	2005/6	2006/7	2007/8	2008/9	2009/10	2010/11	Total	%
***Belgaum***									
NGO Level	16,309	23,053	35,518	20,296	17,508	10,584	8,071	131,339	42.7
SLP Level	0	19,081	4,218	1,895	10,008	12,078	9,595	56,874	18.5
Programme Level	15,381	22,169	17,857	32,860	4,427	10,482	16,321	119,498	38.8
**Total CM & empowerment costs**	**31,690**	**64,304**	**57,593**	**55,051**	**31,942**	**33,144**	**33,986**	**307,711**	100.0
Total HIV prevention costs	256,407	361,204	530,550	1,015,644	763,946	619,066	562,240	4,109,056	
**CM & empowerment costs/HIV prevention costs (%)**	**12.4%**	**17.8%**	**10.9%**	**5.4%**	**4.2%**	**5.4%**	**6.0%**	**8.9%**	
***Bellary***									
NGO Level	8,188	21,807	66,240	51,380	41,548	26,818	22,085	238,065	40.2
SLP Level	-	27,263	5,877	8,960	3,929	6,343	6,071	58,416	9.9
Programme Level	48,397	63,640	63,455	57,239	8,526	20,188	34,975	296,422	50.0
**Total CM & empowerment costs**	**56,585**	**112,711**	**135,572**	**117,578**	**54,004**	**53,349**	**63,131**	**592,903**	**100.0**
Total HIV prevention costs	415,796	502,869	635,771	628,652	297,274	298,942	302,835	3,082,140	
**CM & empowerment costs/HIV prevention costs (%)**	**13.6%**	**22.4%**	**21.3%**	**18.7%**	**18.2%**	**17.8%**	**20.8%**	**19.0%**	

**Table 3:** Unit costs of community mobilisation per person reached by district and year (US$ 2011).

	2004/5	2005/6	2006/7	2007/8	2008/9	2009/10	2010/11	Mean 2004/11
***Belgaum***								
Total CM & empowerment costs	31,690	64,304	57,593	55,051	31,942	33,144	33,986	
NGO CM & empowerment costs	16,309	23,053	35,518	20,296	17,508	10,584	8,071	
Number of persons reached at least once a year	806	2,776	3,143	4,237	2,294	2,679	3,072	
Unit cost per person reached at least once a year	**39**	**23**	**18**	**13**	**14**	**12**	**11**	**19**
NGO level unit cost per person reached at least once a year	**20**	**8**	**11**	**5**	**8**	**4**	**3**	**8**
***Bellary***								
Total CM & empowerment costs	56,585	1,12,711	1,35,572	1,17,552	54,004	53,349	63,131	
NGO CM & empowerment costs	8,188	21,807	66,240	51,380	41,548	26,818	22,085	
Number of persons reached at least once a year	2,741	3,066	4,464	4,157	4,341	5,055	5,743	
Unit costs per person reached at least once a year	**21**	**37**	**30**	**28**	**12**	**11**	**11**	**21**
NGO level costs per person reached at least once a year	**3**	**7**	**15**	**12.4**	**10**	**5**	**4**	**8**

Community mobilisation and empowerment costs contribute between 4.2% to 17.8% in Belgaum and 13.6% to 22.4% in Bellary of total annual programme costs. Bellary shows a more sustained resource commitment for this overall compared to Belgaum; this in parts reflects NGO priorities and also the larger target population of brothel-based workers, which meant that it was feasible to achieve a high attendance at community mobilisation events. In addition Belgaum expanded its more general activities to new areas during the period, which also meant that less attention was able to be placed on supporting community mobilisation. As with the basic HIV prevention package of services, most cost was incurred above the NGO service level for activities such as capacity building, programme management, and support and supervision [52].

The allocation of costs between different activities (Table 4) shows that the majority of the cost was allocated to DICs and events, although this decreased over time. Activities that did not require fixed centres (advocacy and & enabling environment, self-help groups and capacity building for FSWs) required relatively low proportions of total cost, compared to funding for the DICs. The relatively high costs of DICs, was not due to expenditure on buildings, but was due to the costs of holding events in the early years of community mobilisation and empowerment activities. Personnel costs also accounted for a high proportion of the total costs of community mobilisation and empowerment (44% in Belgaum and 32% in Bellary) (Table S4 in Appendix S1).

**Table 4:** Community mobilisation and empowerment costs by activity and year (US$ 2011).

	2004/5	2005/6	2006/7	2007/8	2008/9	2009/10	2010/11	Total	%
***Belgaum***									
Advocacy & Enabling Environment	5,810	11,789	10,559	10,093	55,856	6,076	6,231	56,414	18.3
Drop-in centres and events	17,165	34,831	31,196	29,819	17,302	17,953	18,409	166,677	54.2
Self-help groups and community based organisations	3,169	6,430	5,759	5,505	3,194	3,314	3,399	30,771	10.0
Capacity building and training for FSWs	5,546	11,253	10,079	9,634	5,590	5,800	5,948	53,849	17.5
**Total**	**31,690**	**64,304**	**57,593**	**55,051**	**31,942**	**33,144**	**33,986**	**307,711**	100.0
***Bellary***									
Advocacy & Enabling Environment	9,313	18,550	22,313	19,347	8,888	8,780	10,390	97,582	16.5
Drop-in centres and events	26,053	51,894	62,420	54,123	24,864	24,563	29,067	272,982	46.0
Self-help groups and community based organisations	6,955	13,854	16,664	14,449	6,638	6,557	7,760	72,878	12.3
Capacity building and training for FSWs	14,264	28,412	34,175	29,633	13,613	13,448	15,914	149,461	25.2
**Total**	**56,585**	**112,711**	**135,572**	**117,552**	**54,004**	**53,349**	**63,131**	**592,903**	100.0

The numbers of persons reached at least once a year increases between 2004 and 2011. However in Belgaum it falls in 2008 when the programme is taken over by national authorities in 2008, and also at a time when the programme was beginning to expand to new key populations (high risk men who have sex with men/transgenders); thereafter the numbers of FSWs increases again, but does not rebound to the levels achieved in 2008. The unit cost per FSW reached ranges from US$ 11 to US$ 39 for Belgaum and US$ 11 to US$ 37 in Bellary (Table 4). As with total costs, the highest costs are observed in the first 2–4 years of the intervention – and thereafter costs stabilise at lower levels.

### HIV impact and cost-effectiveness

We estimate that the incremental mean (standard deviation, sd.) impact of community mobilisation and empowerment is 1,256 (308) infections averted in Belgaum, and 2,775 (1,260) infections averted in Bellary in the first seven years of the intervention (Table 5) compared to a situation where no CM and empowerment activities were present. This represents 31% and 39% respectively of the total impact of *Avahan* over this period [29]. Assuming no ART is available to prolong the lives of those in these groups with HIV, we estimate a mean of 20,837 DALYs averted in Belgaum and 46,932 DALYs averted in Bellary as a consequence of the HIV infections averted during the seven year period. In the univariate sensitivity analysis, where ART is available (at a coverage rate of 21–40%) [53], we predict that CM and empowerment would prevent around 383 persons in Belgaum starting treatment and 846 persons in Bellary during this period.

**Table 5:** Mean and standard deviation (sd.)infections averted, cost per infection, DALYs averted, Cost per DALY averted, persons on ART and ART savings, by district and year (U$S2011).

	Infections averted		Cost per infection averted (no ART)		DALYs averted (no ART)		Cost per DALY averted (no ART)		NGO cost per DALY averted (no ART)		Persons on ART averted		ART savings		Cost per DALY averted (including ART savings)	
	Over first 4 years	Over 7 years	Over first 4 years	Over 7 years	Over first 4 years	Over 7 years	Over first 4 years	Over 7 years	Over first 4 years	Over 7 years	Over first 4 years	Over 7 years	Over first 4 years	Over 7 years	Over first 4 years	Over 7 years
**Belgaum**																
Mean	**719**	**1257**	**273**	**234**	**11916**	**20837**	**16.48**	**14.12**	**8.44**	**6.70**	**219**	**383**	424249	741989	Cost Saving	Cost Saving
mean-1.96sd	391	653	137	114	6333	10557	8.28	6.90	4.51	3.44	92	153	29781	41046	Cost Saving	Cost Saving
mean+1.96sd	1046	1860	408	353	17499	31118	24.67	21.33	12.38	9.96	346	613	818718	1442933	9.42	Cost Saving
**Bellary**																
Mean	**1332**	**2775**	**323**	**228**	**22533**	**46932**	**19.12**	**13.48**	**7.80**	**6.22**	**406**	**846**	787793	1642173	Cost Saving	Cost Saving
mean-1.96sd	247	305	27	3	4035	4843	1.53	0.15	0.87	0.21	43	28	-163829	-449356	Cost Saving	Cost Saving
mean+1.96sd	2417	5246	619	452	41031	89021	36.71	26.82	14.73	12.22	769	1664	1739414	3733701	18.88	10.97

Bringing together this incremental impact with incremental cost we estimate a mean (sd.) incremental cost per infection averted of US$ 234 (61) in Belgaum and US$ 228 (114) in Bellary. Assuming no ART this results in a mean (sd.) incremental cost per DALY averted of US$ 14.12 (3.68) in Belgaum and US$ 13.48 (6.80) in Bellary. Exploring the inclusion of ART in a sensitivity analysis we find that if ART cost savings are included, the intervention becomes cost saving. The sensitivity analysis around discount rates did not increase the incremental cost per DALY above the WTP threshold (Figure S1 in Appendix S1).

## Discussion

Our findings suggest that investment in CM and empowerment, as a critical enabler of basic HIV prevention services aimed at FSWs, is cost-effective; may even be cost saving if future ART savings are taken into account and therefore are good ‘value for money’. While some may be concerned that additional expenditure on CM and empowerment has an opportunity cost in terms of other HIV prevention and treatment activities forgone (given current resource constraints on HIV programmes), the very low incremental cost-effectiveness ratio of CM and empowerment suggests that this is unlikely - and that CM and empowerment should be considered as an integral component of HIV prevention programming for FSWs [16], [18]. In this sense, CM and empowerment can be argued to be as much of an essential HIV prevention activity for FSWs as many other HIV interventions labeled as ‘core’ HIV prevention services.

Moreover our findings are likely to be conservative from a broader economic welfare perspective as our analysis focuses on outcomes in terms of the HIV prevention benefits alone. We have excluded other health benefits that may arise from this type of intervention, such as reductions in violence and alcohol use [11]. We have also not examined possible impact on ART uptake or adherence. Beyond these health benefits, there may be broader benefits derived in terms of economic welfare (access to income generation schemes) and poverty reduction objectives (access of this very vulnerable group to social entitlements and education for their children) [12]. Therefore, while our results suggest that investment in community mobilization and empowerment is justifiable from the HIV perspective alone, exploring wider economic and health benefits further may also justify funding from those with a broader development perspective – at a time when HIV funding is increasingly required to find its place in the broader post-Millennium Development Goals context.

For those interested in broader social welfare as their primary funding objective, it is important to consider the added value of making the most efficient use of the infrastructure developed by HIV prevention programmes such as *Avahan* to reach many vulnerable population groups who may not have previously been reached at this scale by other development efforts. From a poverty reduction perspective, the potential of incurring relatively low incremental costs to expand development efforts to improve the welfare of FSWs more generally may be an opportunity not to be missed – and thus greater synergies between traditional HIV funders and development funders need to be explored.

The high cost-effectiveness of community mobilization and empowerment found is likely to be primarily due to the underlying cost-effectiveness of HIV prevention in India [20], [22], [54]. The low cost of HIV prevention in India [55], together with a sizeable impact on the HIV epidemic [29], means that community mobilization and empowerment, as an intertwined intervention that enhances the effectiveness of the HIV prevention, has a sound basis on which to yield substantial returns from an HIV perspective. However, in environments where HIV prevention targeted at key populations is less cost-effective, it remains to be seen whether the same results as we find here could be achieved. In India the costs of CM and empowerment range between 10–20% of the total HIV prevention cost; and the incremental unit cost per person reached a year is around US$ 11, which may be considered affordable within the current National Aids Control Organisation expenditures of around US$ 50 per person reached. But this may not be the case in other settings. There are a number of key contextual factors that are likely to impact on the likely transferability of our findings to other settings. These include the legal context of sex work – for example, where sex work is highly criminalised, it may be far more difficult to implement sex worker mobilisation interventions, or they may require a different combination of intervention inputs. Other contextual issues include the acceptability to the community of the specific intervention models evaluated here, existing levels of sex worker empowerment and organisation, socio-cultural norms and the characteristics of sex work in different settings.

Our results may also be viewed by some as limited, even with the setting studied, as the analysis of the causal pathway relies on a conceptual framework, observational data and model-based evidence of effectiveness, rather than on experimental evidence. This approach provides plausible evidence of effect [29]; and is feasible in settings where experimental designs are not appropriate [56]. However, while we adjusted for socio-demographic confounders in our exposure analysis, and carefully constructed our control group in the modelling, our results may still be limited by some selection bias, in that we may not have entirely removed endogeneity in the relationship between FSW behaviour related to HIV prevention and their involvement in community mobilisation. In addition, our approach allows estimation of the impact of CM and empowerment activities including reduced onwards transmission from FSWs who were infected prior to engaging with CM and empowerment. Through the combination of Bayesian methods and a probabilistic sensitivity analysis around the incremental cost-effectiveness ratio we also attempt to provide a realistic and transparent presentation of the uncertainty inherent in this type of analysis. In particular, our estimates of infections averted have high standard deviations, primarily due to uncertainty around the estimated population size studied [29]. But nevertheless we find our results to be robust.

As an economic evaluation, our study is also limited in that the strength of much of our evidence is based on data from costs and effects which are derived from a single study setting. We chose this approach due to the dearth of data on costs and effects of community mobilisation and empowerment (from the same setting) globally. However, our findings on effects are supported by a recent meta-analysis of CM and empowerment interventions [57]. Finally, our study is limited in the fact that it takes a provider rather than a societal perspective, and thus excludes the costs that FSWs and other providers may incur and contribute towards any intervention effect. This may mean we over-estimate cost-effectiveness. However, given the very low incremental cost-effectiveness ratio, this is highly unlikely to impact our main findings and inference above. Nevertheless, given the local nature of our study, and these limitations, we strongly suggest that further research across a number of settings is conducted to bring added precision and confirmation to our findings.

Overall, our findings provide strong evidence of the value of investment in sex worker community mobilization, and lead us to recommend the sustained funding of community mobilisation and empowerment activities as part of HIV prevention for FSWs in India, and the consideration of funding for scaling up similar interventions in other contexts, (accompanied with a careful evaluation of impact and cost-effectiveness during scale-up). The potential for multiple HIV, development and poverty reduction benefits for a low cost are an important element of such investments, meriting further research, as part of a broader, developmental perspective on future investments. Finally, our findings suggest that community mobilisation for especially at risk and vulnerable populations play a central role in the HIV response, and merit consideration by HIV programme managers and their funders, as central components of the core package of HIV prevention for FSWs.

## Supporting Information

Appendix S1 **Text S1,** Summary of Exposure Analysis. **Table S1,** Peer education and service uptake, risk and HIV/STI prevalence, adjusted for socio-demographic characteristics and community mobilisation (IBBA Round 1 (2005), pooled data from 4 districts). **Table S2,** Peer education and power, service uptake, risk and HIV/STI prevalence, adjusted for socio-demographic characteristics and community mobilisation (IBBA 2011 and STI data 2008, pooled data from 4 districts). **Table S3,** Impact and cost-effectiveness model parameters. **Table S4,** NGO* and SLP** community mobilisation economic costs by input and year (US$ 2011). **Figure S1,** Acceptability Curves**. Figure S2,** FSW HIV prevalence (%) by year in four districts used in the exposure analysis. **Figure S3,** Condom use by year in four districts used in the exposure analysis.
(DOCX)
