# Supplementary material for: Community Mobilisation and Empowerment Interventions as Part of HIV Prevention for Female Sex Workers in Southern India: A Cost-Effectiveness Analysis
Source: PLoS One. 2014 Oct 21;9(10):e110562. doi: 10.1371/journal.pone.0110562 (PMC4204894; doi:10.1371/journal.pone.0110562)
Supplement: Appendix S1 — Text S1, Summary of Exposure Analysis. Table S1, Peer education and service uptake, risk and HIV/STI prevalence, adjusted for socio-demographic characteristics and community mobilisation (IBBA Round 1 (2005), pooled data from 4 districts). Table S2, Peer education and power, service uptake, risk and HIV/STI prevalence, adjusted for socio-demographic characteristics and community mobilisation (IBBA 2011 and STI data 2008, pooled data from 4 districts). Table S3, Impact and cost-effectiveness model parameters. Table S4, NGO* and SLP** community mobilisation economic costs by input and year (US$ 2011). Figure S1, Acceptability Curves. Figure S2, FSW HIV prevalence (%) by year in four districts used in the exposure analysis. Figure S3, Condom use by year in four districts used in the exposure analysis. (DOCX) [file pone.0110562.s001.docx]

## Supplementary Appendix

**Text S1 – Summary of Exposure Analysis**

We performed a secondary analysis of the FSW IBBA surveys pooled across four districts in Karnataka (Belgaum and Bellary, Bangalore and Shimoga). Data from the four districts was pooled for each survey round to enable sufficient statistical power, and we analysed data from the baseline (R1) and final survey rounds (R3). Two exposure variables were used for this study: Exposure to a peer educator and exposure to CM activities (defined as: ‘high’ if a woman reported being a member of a FSW peer group and/or FSW collective, ‘medium’ if she had ever attended a drop-in centre and/or a non-governmental organisation (NGO) meeting and ‘low’ if she had not done any of the above). The primary outcome used here was ‘condom use at last sex with occasional clients’ with ‘occasional’ clients defined as “clients who have come to you only once or a few times, but you do not remember their faces or do not know them”, and ‘repeat’ clients defined as “clients you recognise well, who have come to you repeatedly and you know them”. All statistical analyses were performed using the survey data analysis techniques in STATA, version 12.0 as previously described (Beattie et al CM paper). Odds ratios (OR) were used as the measure of association, and the Wald chi-squared test was the statistical test used. Logistic regression analyses were used to examine associations between exposure to CM and the various outcome variables. In the multivariate models, potential socio-demographic and sex work characteristic confounders were added to the model using a stepwise approach. Those which caused the OR of the independent variable to change by 10% or more were included in the final model. The adjusted Wald test was used to test for effect modification for the following socio-demographic variables: age, duration in sex work, place where solicit clients, place where entertain clients, charge per sex act, and regular partner. To explore the hierarchical relationships between different protective factors for HIV infection among FSWs, pathway analyses were used to obtain estimates of the degree to which CM may be having an impact on key HIV related outcomes (including condom use at last sex with occasional clients), over and above peer education activities alone [58], as previously described. For this, we first used logistic regression to examine associations between peer education and the outcome variables using data from R3. We then added CM to the adjusted multivariate model, to assess whether, by adjusting for CM, the adjusted ORs substantially shifted closer to 1.0 (which would suggest that CM was acting on the causal pathway between peer education and the outcome variable) [58]. When peer education was significantly associated with an outcome variable, we calculated the proportion difference in the adjusted ORs between peer education and the outcome variable and peer education and the outcome variable, adjusting for CM ((OR adjusted for confounders – OR adjusted for confounders and CM)/ OR adjusted for confounders) (Last column of tables S2-S3).

**Table S1: Peer education and service uptake, risk and HIV/STI prevalence, adjusted for socio-demographic characteristics and community mobilisation (IBBA Round 1 (2005), pooled data from 4 districts).**

|  |  | Not met a peer educator  (N=210) % | Met a peer educator  ( n=1673) % | P value  (Wald test) | Crude OR | OR adjusted for sociodemographic confounders^1^ | OR adjusted for sociodemographic confounders & CM^2^ | | Proportional difference in OR (%)^3^ |
| --- | --- | --- | --- | --- | --- | --- | --- | --- | --- |
| **HIV/STI service uptake** | Visited project STI clinic past 6 months | 1.1 | 79.9 | <0.001 | 368.13*** (105.32, 1286.77) | 608.20*** (139.24, 2656.58) | 132.53*** (26.40, 665.30) | | 78.2 |
|  | Ever taken an HIV test | 16.9 | 30.2 | 0.0004 | 2.13*** (1.39, 3.25) | 1.92** (1.25, 2.96) | 1.13 (0.66, 1.93) | | 41.1 |
| **Condom use last sex** | Occasional client^4^ | 72.7 | 89.2 | <0.001 | 3.09*** (2.00, 4.77) | 2.86*** (1.78, 4.58) | 1.54 (0.86, 2.75) | 46.2 | |
|  | Repeat client^5^ | 62.2 | 78.8 | 0.0001 | 2.26*** (1.49, 3.40) | 1.72** (1.14, 2.58) | 1.00 (0.62, 1.62) | 41.9 | |
|  | Regular partner | 30.0 | 41.0 | 0.063 | 1.62 (0.97, 2.70) | 1.24 (0.70, 2.18) | 0.78 (0.40, 1.51) |  | |
| **Condom use always** | Occasional client^4^ | 50.8 | 77.5 | <0.001 | 3.34*** (2.38, 4.67) | 2.54*** (1.71, 3.77) | 1.46 (0.93, 2.30) | 42.5 | |
| **HIV/ STI prevalence** | HIV | 8.3 | 19.5 | 0.0007 | 2.65** (1.48, 4.76) | 1.74 (0.88, 3.47) | 2.09* (0.98, 4.47) |  | |
|  | Gonorrhoea | 4.2 | 2.9 | 0.41 | 0.68 (0.27, 1.71) | 0.39* (0.16, 0.95) | 0.49 (0.17, 1.40) | -25.6 | |
|  | Chlamydia | 6.3 | 5.6 | 0.71 | 0.88 (0.45, 1.72) | 0.98 (0.45, 2.10) | 1.18 (0.49, 2.88) |  | |
|  | Gonorrhoea &/or Chlamydia | 8.8 | 7.9 | 0.71 | 0.89 (0.48, 1.65) | 0.86 (0.43, 1.71) | 1.05 (0.48, 2.30) |  | |
|  | Active syphilis | 6.5 | 7.4 | 0.75 | 1.16 (0.48, 2.80) | 0.95 (0.39, 2.34) | 0.86 (0.33, 2.25) |  | |
|  | High-titre syphilis | 3.7 | 4.3 | 0.82 | 1.15 (0.34, 3.84) | 1.16 (0.36, 3.80) | 1.16 (0.31, 4.35) |  | |
|  | HSV-2 | 61.0 | 72.1 | 0.0026 | 1.65** (1.19, 2.29) | 1.23 (0.86, 1.74) | 1.13 (0.75, 1.72) |  | |

^1^ Models adjusted for duration in sex work, sex work typology, district, marital status, additional source of income to sex work, migrant sex worker (sold sex outside district in past 6 months) and regular partner. ^2^Models adjusted for sociodemographic confounders and community mobilisation. ^3^Proportional difference in OR calculated for outcome variables significantly associated with peer education in multivariate analyses: ((OR adjusted for confounders – OR adjusted for confounders and CM)/ OR adjusted for confounders). ^4^‘Occasional’ client defined as “client who has come to you only once or a few times but you do not remember their face or do not know them”. ^5^‘Repeat’ client defined as “client you recognise well, who has come to you repeatedly and you know them”. *p<0.05; **p<0.01; ***p<0.0001.

**Table S2. Peer education and power, service uptake, risk and HIV/STI prevalence, adjusted for socio-demographic characteristics and community mobilisation (IBBA 2011 and STI data 2008, pooled data from 4 districts).**

|  |  | Not met a peer educator  (N=48) % | Met a peer educator  ( n=1886) % | P value  (Wald test) | Crude OR | OR adjusted for sociodemographic confounders^1a^ | OR adjusted for sociodemographic confounders & CM^2^ | Proportional difference in OR (%)^3^ |
| --- | --- | --- | --- | --- | --- | --- | --- | --- |
| **Power** | Feel a sense of unity with other FSWs | 60.8 | 93.5 | <0.001 | 9.32**  (5.02, 17.33) | 8.14**  (4.22, 15.71) | 4.38**  (2.01, 9.56) | 46.2 |
|  | Negotiated on behalf of other FSWs | 0 | 21.5 | 0.0047 | - | - | - |  |
|  | Attended a public event to campaign for the rights of FSWs | 0 | 13.0 | 0.035 | - | - | - | - |
|  | In past month had time when wanted to use a condom with a client but did not (self-efficacy) % ^4^ | 66.0 | 87.7 | 0.0001 | 3.69** (1.84, 7.38) | 4.66**(2.29, 9.46) | 3.48* (1.41, 8.60) | 25.3 |
| **HIV/STI service uptake** | Visited project STI clinic past 6 months | 0 | 85.5 | <0.001 | - | - | - |  |
|  | Ever taken an HIV test | 38.7 | 95.6 | <0.001 | 34.68** (16.54, 72.72) | 28.81** (13.48, 61.57) | 6.32** (2.67, 15.00) | 78.1 |
| **Condom use last sex** | Occasional client^5^ | 73.1 | 95.9 | <0.001 | 8.67** (4.05, 18.56) | 10.96** (5.00, 24.01) | 6.78* (1.96, 23.44) | 38.1 |
|  | Repeat client^6^ | 72.4 | 92.2 | <0.001 | 4.50** (2.07, 9.78) | 5.58** (2.49, 12.48) | 2.40 (0.80, 7.24) | 57.0 |
|  | Regular partner | 15.5 | 39.7 | 0.0010 | 3.59* (160, 8.08) | 7.41** (2.81, 19.56) | 5.50* (1.74, 17.35) | 25.8 |
| **Condom use always** | Occasional client^5^ | 63.9 | 91.7 | <0.001 | 6.19** (0.96, 12.94) | 6.16** (2.75, 13.84) | 3.32* (1.17, 9.40) | 46.1 |
| **HIV/ STI prevalence** | HIV | 10.9 | 10.8 | 0.98 | 0.99  (0.41, 2.42) | 0.95  (0.33, 2.76) | 0.82  (0.24, 2.75) |  |
|  | Gonorrhoea^1b^ | 7.2 | 2.3 | 0.0014 | 0.30*  (0.14, 0.65) | 0.56  (0.25, 1.24) | 0.68  (0.21, 2.22) |  |
|  | Chlamydia^1b^ | 15.9 | 5.4 | <0.001 | 0.30**  (0.18, 0.51) | 0.78  (0.46, 1.33) | 1.15  (0.64, 2.07) |  |
|  | Gonorrhoea &/or Chlamydia^1b^ | 18.5 | 6.8 | <0.001 | 0.32**  (0.19, 0.53) | 0.75  (0.45, 1.25) | 1.18  (0.67, 2.08) |  |
|  | Active syphilis | 7.3 | 4.5 | 0.38 | 0.60  (0.19, 1.92) | 0.69  (0.21, 2.20) | 0.70  (0.21, 2.37) |  |
|  | High-titre syphilis | 2.1 | 1.7 | 0.85 | 0.83  (0.12, 5.56) | 1.20  (0.17, 8.68) | 2.06  (0.18, 24.03) |  |

^1a^Models adjusted for duration in sex work, sex work typology, district, marital status, localite (born in district) and repeat clients ^1b^Data presented from 2008. Models adjusted for localite (born in district), additional source of income to sex work, regular partner, duration in sex work, sex work typology (where solicit clients) and district. ^2^Models adjusted for sociodemographic confounders and community mobilisation. ^3^ Proportional difference in OR calculated for outcome variables significantly associated with peer education in multivariate analyses: ((OR adjusted for confounders – OR adjusted for confounders and CM)/ OR adjusted for confounders). ). ^4^Self-efficacy defined as saying ‘no’ to: ‘in the past month, was there a time when you wanted to use a condom with a client but did not use it’. ^5^‘Occasional’ client defined as “client who has come to you only once or a few times but you do not remember their face or do not know them”. ^6^‘Repeat’ client defined as “client you recognise well, who has come to you repeatedly and you know them”. *p<0.05; **p<0.01; ***p<0.0001.

**Impact and cost-effectiveness parameters**

**Table S3: Impact and cost-effectiveness model parameters**

*Ranges for key biological parameters used in model*

| **Definition of model input** | **Model inputs** | **Reference** |
| --- | --- | --- |
| ***HIV model parameters*** |  |  |
| ***Average duration of HIV stages (months):*** |  | Based on [59,60]. |
| **Early HIV high viraemia phase** | 2-6 |  |
| **Between initial high viraemia and pre-AIDS** | 70-91 |  |
| **Pre-AIDS high viraemia phase** | 6-18 |  |
| **Duration of AIDS phase without treatment** | 11.6-29.4 |  |
| **Probability of HIV transmission per sex act in asymptomatic stage male-to-female / female-to-male** | 0.06-0.11% / 0.01-0.14% | Reviewed in [61] |
| **HIV transmission multiplicative RR per sex act from early / pre-AIDS high viraemia phases** | 4.5-18.8 / 4.5-11.9 | From [61-63] |
| **Probability of HSV-2 transmission per sex act in latent/asymptomatic shedding stage (male to female)** | 0.05-0.20% | [64,65] |
| **Ratio HSV-2 male-to-female: female-to-male transmission** | 2-5 | [64,65] |
| **Probability of male-to-female syphilis transmission per sex act** | 0.03-0.2 | Based on [66] |
| **Ratio of syphilis transmission probabilities female to male: male to female** | 0.33-1.0 | Based on [66] |
| **Per-act condom effectiveness against HIV/HSV-2 and syphilis** | 0.8-0.95/0.4-0.7 | [67,68]/[64,69] |

*Ranges for key behavioural parameters and prevalence data used in the model*

| **Parameter** | **Belgaum** | **Bellary** |
| --- | --- | --- |
| **Size of FSW population** | 1440-2170 | 2850-4270 |
| ***Condom use parameters*** |  |  |
| **Annual % increase in condom use before *Avahan*** | 3.27-5.67% | 3.90-6.54% |
| ***% of FSW in the high condom use group under intervention scenario:*** |  |  |
| - **At the start of *Avahan*** | 42.2-54.7 | 29.7-47.9 |
| - **At IBBA round 1** | 87.2-94.5 | 66.8-78.7 |
| - **At IBBA R2** | 85.6-92.6 | 85.4-92.8 |
| - **At IBBA R3** | 94.6-98.7 | 82.9-91.9 |
| **% of change in condom use from *Avahan* due to CM & empowerment activities in round 1 / round 3** | 46.2% / 38.1% | 46.2% / 38.1% |
| **Proportion of FSW who are lower activity** | 0.47-0.62 | 0.44-0.60 |
| ***Total number of clients per month for lower/higher activity FSWs at:*** |  |  |
| **- IBBA round 1** | 21.0-25.5 / 90.0-113.0 | 12.1-15.9 / 70.1-101.0 |
| **- IBBA round 2** | 25.5-28.0 / 83.2-97.4 | 25.3-28.8 / 98.2-169.0 |
| **- IBBA round 3** | 19.6-22.0 / 66.8-84.7 | 16.6-18.3 / 55.2-91.1 |
| **Average time spent in sex work if lower/higher activity** | 149.0-202.0 / 99.4-131.0 | 105.0-136.0 / 86.0-111.0 |
| ***Client parameters*** |  |  |
| **Proportion of clients who are lower activity** | 0.58-0.69 | 0.40-0.54 |
| **Duration as clients by activity level (lower / higher activity)** | 95-120 / 106-145 | 91-119 / 112-147 |
| **Number of FSW visited per month by activity level (lower / higher activity)** | 1.0 / 2.3-2.8 | 1.0 / 2.4-2.7 |
| **% of clients who have long-term partner by time buying sex (0-4 years / 5+ years buying sex)** | 32.4-49.8 / 80.9-90.7 | 36.9-59.1 / 82.5-92.6 |
| ***% prevalence data for fitting*** |  |  |
| **FSW round 1 HIV** | 33.9 (27.6-40.2) | 15.7 (11.1-20.0) |
| **FSW round 1 HSV** | 83.9 (78.6-89.1) | 70.2 (63.7-76.8) |
| **FSW round 1 Syphilis** | 3.3 (1.5-5.1) | 2.0 (0.7-3.4) |
| **Client round 1 HIV** | 6·2 (3.6-8.8) | 6.0 (2.6-9.5) |
| **Client round 1 HSV** | 27.6 (23.3-32.3) | 25.8 (20.7-31.0) |
| **Client round 1 Syphilis** | 1.9 (0.5-3.3) | 2.3 (0.0-2.9) |
| **FSW prevalence ratio round 2:round 1** | 0.58-1.02 | 0.62-1.25 |
| **FSW prevalence ratio round 3:round 1** | 0.57-0.98 | 0.28-0.72 |

*Ranges of key parameters used in the estimation of DALYS*

| **Parameter** | **Sampling distribution** | **Source** |
| --- | --- | --- |
| ***Age at HIV infection (years)*** |  |  |
| **- FSWs in Belgaum** | Uniform(29.5,32.1) | IBBA data |
| **- FSWs in Bellary** | Uniform(26.9,30.7) |  |
| **- Clients/general population in Belgaum** | Uniform(31.5,35.6) |  |
| **- Clients/general population in Bellary** | Uniform(31.5,35.7) |  |
| **DALY weight for late-stage HIV infection** | Truncated N(0.23,0.0018) | [70] |
| **DALY weight for AIDS** | Truncated N(0.55,0.0072) | [70] |
| **DALY weight on ART** | Truncated N(0.057,0.00013) | [70] |
| ***Life expectancy without HIV by age group:*** | Point estimates | WHO life |
| **- 25-29** | 43.8 | expectancy tables |
| **- 30-34** | 39.3 | (2009) |
| **- 35-39** | 34.9 |  |
| **- 40-44** | 30.7 |  |
| **- 45-49** | 26.5 |  |
| **- 50-54** | 22.6 |  |
| **Unit costs of ART (US$ 2011)** | Triangular(200,400,600) | [71] |
| **Extra life expectancy on ART if HIV positive (years)** | Uniform(2.50,8.00) | [60,72] |
| **Coverage of ART of eligible individuals in India** | Uniform (0.21,0.40) | [73] |

**Costing**

**Table S4: NGO* and SLP** community mobilisation economic costs by input and year (US$ 2011)**

|  | **2004/5** | **2005/6** | **2006/7** | **2007/8** | **2008/9** | **2009/10** | **2010/11** | **Total** | **%** |
| --- | --- | --- | --- | --- | --- | --- | --- | --- | --- |
| ***Belgaum*** |  |  |  |  |  |  |  |  |  |
| **Total capital costs** | **817** | **1,710** | **1,448** | **1,130** | **1,280** | **1,594** | **1,841** | **9,820** | 5 |
| Personnel | 8,224 | 11,993 | 14,452 | 8,738 | 15,775 | 14,330 | 9,135 | 82,646 | 44 |
| Travel | 936 | 2,133 | 2,369 | 1,656 | 2,299 | 1,833 | 1,578 | 12,803 | 7 |
| Building operating & maintenance | 4,605 | 3,629 | 2,938 | 1,080 | 930 | 721 | 1,117 | 15,019 | 8 |
| Materials and supplies/ event costs | 1,450 | 22,515 | 17,307 | 8,987 | 6,465 | 3,840 | 3,648 | 64,212 | 34 |
| Other overheads | 277 | 156 | 1,224 | 601 | 766 | 344 | 347 | 3,715 | 2 |
| **Total recurrent costs** | **15,492** | **40,425** | **38,288** | **21,062** | **26,235** | **21,068** | **15,825** | **178,395** | **95** |
| **Grand Total** | **16,309** | **42,135** | **39,736** | **22,191** | **27,515** | **22,662** | **17,666** | **188,213** | **100** |
|  |  |  |  |  |  |  |  |  |  |
| ***Bellary*** |  |  |  |  |  |  |  |  |  |
| **Total capital costs** | **1,034** | **1,975** | **4,218** | **2,899** | **1,844** | **2,243** | **2,375** | **16,588** | **6** |
| Personnel | 2,820 | 11,431 | 18,512 | 14,308 | 16,312 | 19,407 | 13,233 | 96,023 | 32 |
| Travel | 256 | 1,561 | 2,006 | 2,429 | 1,660 | 2,774 | 2,065 | 12,751 | 4 |
| Building operating & maintenance | 213 | 1,061 | 345 | 524 | 1,517 | 1,497 | 1,596 | 6,753 | 2 |
| Information Education Communication supplies | 3,866 | 1,661 | 3,467 | 2,722 | 430 | 1,233 | 588 | 13,968 | 5 |
| Materials and supplies/ event costs | 0 | 31,110 | 41,701 | 36,312 | 22,877 | 5,419 | 7,739 | 145,157 | 49 |
| Other overheads | 0 | 271 | 1,866 | 1,119 | 837 | 586 | 560 | 5,239 | 2 |
| **Total recurrent costs** | **7,154** | **47,095** | **67,898** | **57,415** | **43,633** | **30,917** | **25,781** | **279,893** | **94** |
| **Grand Total** | **8,188** | **49,070** | **72,117** | **60,313** | **45,477** | **33,160** | **28,156** | **296,481** | **100** |
|  |  |  |  |  |  |  |  |  |  |

***Non-governmental organisation , **State Lead Partner**

**Figure S1: Acceptability Curves**

**Belgaum**

**Bellary**

**Figure S2: FSW HIV prevalence (%) by year in four districts used in the exposure analysis**

**Figure S3: Condom use by year in four districts used in the exposure analysis**

# References
